# Microbial life in 25-m-deep boreholes in ancient permafrost illuminated by metagenomics

**DOI:** 10.1186/s40793-023-00487-9

**Published:** 2023-04-13

**Authors:** Xiaofen Wu, Abraham L. Almatari, Wyatt A. Cyr, Daniel E. Williams, Susan M. Pfiffner, Elizaveta M. Rivkina, Karen G. Lloyd, Tatiana A. Vishnivetskaya

**Affiliations:** 1 Center for Environmental Biotechnology, University of Tennessee, 1416 Circle Drive, Knoxville, TN 37996-1605 USA; 2 Soil Cryology Laboratory, Institute of Physicochemical and Biological Problems in Soil Science, Russian Academy of Sciences, Pushchino, Russia 142290; 3 Department of Microbiology, University of Tennessee, Knoxville, TN 37996 USA

**Keywords:** Arctic, Permafrost, Metagenomics, Metagenome-assembled genomes, Metabolic potential

## Abstract

This study describes the composition and potential metabolic adaptation of microbial communities in northeastern Siberia, a repository of the oldest permafrost in the Northern Hemisphere. Samples of contrasting depth (1.75 to 25.1 m below surface), age (from ~ 10 kyr to 1.1 Myr) and salinity (from low 0.1–0.2 ppt and brackish 0.3–1.3 ppt to saline 6.1 ppt) were collected from freshwater permafrost (FP) of borehole AL1_15 on the Alazeya River, and coastal brackish permafrost (BP) overlying marine permafrost (MP) of borehole CH1_17 on the East Siberian Sea coast. To avoid the limited view provided with culturing work, we used 16S rRNA gene sequencing to show that the biodiversity decreased dramatically with permafrost age. Nonmetric multidimensional scaling (NMDS) analysis placed the samples into three groups: FP and BP together (10–100 kyr old), MP (105–120 kyr old), and FP (> 900 kyr old). Younger FP/BP deposits were distinguished by the presence of Acidobacteriota, Bacteroidota, Chloroflexota_A, and Gemmatimonadota, older FP deposits had a higher proportion of Gammaproteobacteria, and older MP deposits had much more uncultured groups within Asgardarchaeota, Crenarchaeota, Chloroflexota, Patescibacteria, and unassigned archaea. The 60 recovered metagenome-assembled genomes and un-binned metagenomic assemblies suggested that despite the large taxonomic differences between samples, they all had a wide range of taxa capable of fermentation coupled to nitrate utilization, with the exception of sulfur reduction present only in old MP deposits.

**Supplementary Information:**

The online version contains supplementary material available at 10.1186/s40793-023-00487-9.

## Introduction

Permafrost is a unique perennially frozen environment that covers ~ 24% of the land surface area in Earth's Northern Hemisphere [1]. Permafrost temperature varies from around − 2 °C in Svalbard [2] and near the southern permafrost boundary in Siberia to the lower temperature of − 18 °C on Ellesmere Island, Canada [3]. Permafrost deposits are heterogenous and their physicochemical properties are determined by their origin (e.g., lacustrine and alluvial versus marine), formation (e.g., epigenetic or consecutively frozen versus syngenetic or simultaneously frozen) and age since sediments have been frozen (e.g., climate history indicates that perennially frozen ground formed and thawed repeatedly leading to layering from young to older permafrost) [4, 5]. Permafrost temperature and size of mineral particles (e.g., clay, silt or sand) affect the amount and thickness of unfrozen water films, which typically accounts for 1.5–7% of total water [6]. Unfrozen water may form thicker films and brine lenses (cryopegs) with free supercooled saline brine with mineralization up to 250 g l^−1^ and low water activity [7]. The perennially frozen deposits in the Arctic store large amounts of soil organic carbon [8–10], however frozen sequestered carbon is not very available as a nutritional substrate within the frozen ground. The preserved soil organic carbon is potentially vulnerable to remobilization and becomes accessible to microbial degradation as a result of the permafrost thawing due to increased global temperatures [11].

Despite the freezing temperatures, insignificant content of liquid water, low water activity, and nutrient deprivation, viable and at times metabolically active microorganisms are confirmed to be present in permafrost. Several lines of evidence support this statement, including (1) isolation of viable culturable cells [12]; (2) observation of viable non-culturable cells using LIVE/DEAD differential staining coupled with fluorescence microscopy [13, 14]; (3) demonstration of biomass production and genome replication of permafrost microorganisms at negative temperatures though radioactively labeled isotope-probing [15, 16]; (4) observation of viable and metabolically active cells using aspartic acid racemization [17]; (5) revealing metabolic pathways involved in microbial adaptations to permafrost conditions [18]; and (6) indication of metabolic activity and cell division based on metatranscriptomes and metaproteomes in intact permafrost [19]. A metagenomic study of the late Pleistocene Siberian permafrost samples showed that microbial community composition depends on the conditions existed at time of permafrost formed [20]. The ~ 30 kyr old ice-rich syngenetically frozen sediments accumulated under cold arid aerobic conditions showed absence of detectable methane and a low abundance of methanogenic archaea and genes encoding carbon and nitrogen related functions but a higher abundance of genes associated with sulfur cycling [20]. Contrasting data were shown for the ~ 30 kyr old floodplain lake-alluvial sediments, which had formed epigenetically in anaerobic conditions and exhibited a presence of 1.2 mmol kg^−1^ biogenic methane and up to 1.5% of methanogenic Archaea [20]. Cryopegs encapsulated in ancient permafrost marine sediments epigenetically frozen ~ 200 kyr ago demonstrated through cultivation studies the existence of indigenous microbial communities [21]. Permafrost microbes surrounded by microscopic layers of liquid brines develop adaptations to this environment in energy production and conversion and carbohydrate transport and metabolism as shown through comparative analyses of metagenome-assembled genomes (MAGs) derived from samples of 20 kyr to ~ 1 Myr old permafrost with genomes of non-permafrost counterparts [18]. The study of *Psychrobacter articus* 273-4 isolated from 40 kyr old permafrost showed that the two-component regulation system, DnaK suppressor, HSP70, peptidylprolyl isomerase, FKBP-type, and other translation and metabolism-related proteins play important roles in the adaptation to salinity [22].

Increases in salinity and consecutive decrease in water potential of permafrost deposits will cause a formation of specialized microbial communities due to changes in population structure and cellular adaptation in order to withstand salinization [22, 23]. Soil salinity is a key determinant of microbial community composition [24], so permafrost with naturally differing salinities is likely to harbor different microbial communities. Salt adapted microbial communities may affect the degradation of organic matter and emission of greenhouse gasses such as carbon dioxide (CO_2_), methane (CH_4_), and nitrous oxide (N_2_O) [25]. Study of agricultural soils of different salinities showed that high salinity decreases bacterial phylogenetic diversity, destabilizes bacterial networks and inhibits key nitrogen metabolisms [26]. However, the impact of salinity on permafrost bacterial community composition and community-level function remains uncertain. The current dataset provides a unique opportunity to compare the long-term impact of negative temperature and varying salinity on permafrost microbial communities. Characterization of microbial communities and their metabolic potential from diverse permafrost deposits becomes of ultimate importance due to global warming and increasing permafrost thawing. The latter process will result in release of frozen microbes and unleashing of microbial processes that in turn will contribute to the decomposition of sequestered organic carbon, global flux of greenhouse gasses and other consequences, for example, release of mercury from thawing permafrost [27].

In this research we studied pristine and unique samples collected from epigenetically formed permafrost strata in northeastern Siberia. The permafrost in that area developed during cold periods in the Late Pliocene, Pleistocene and Holocene epochs and extends to hundreds of meters in depth forming layers of different ages and different origin, e.g., freshwater, brackish and marine [4, 28]. The deep layers represent one of the oldest continuously frozen localities on Earth [7]. Therefore, permafrost deposits of northeastern Siberia that did not thaw during the Holocene climatic optimum are of great interest for the microbiology and microbial ecology research. Much of our knowledge about Siberian permafrost is derived from cultivation studies, which revealed communities of anaerobic and aerobic, spore-forming and non-spore-forming bacteria, green algae, yeast, fungi as well as highly specialized organisms like methanogenic archaea [12, 29, 30]. Unlike culture-dependent methods, which uncover limited diversity, and 16S rRNA gene sequencing, which identifies the taxonomic composition of bacterial and archaeal communities, metagenomic sequencing allows the structural characterization of the whole microbial communities and their metabolic potential.

We used both 16S rRNA gene amplicon and metagenome sequencing to investigate the taxonomic composition, genetic diversity and metabolic potential of permafrost microbial communities in samples of epigenetically frozen sediments along a salinity gradient. The northeastern Siberia permafrost samples were from different depths (1.75–25.1 m), ages (~ 10 kyr–1.1 Myr) and salinity types (freshwater, brackish and marine permafrost). The metagenome data were assembled and partitioned into MAGs to reconstruct metabolic pathways for the dominant members of the microbial communities. To complement the genome-based metagenomics, a gene-centric analysis was performed by direct examination of un-binned and assembled metagenomic data.

## Materials and methods

### Sampling sites and collection of samples

The sampling sites were located within the Kolyma Lowland in northeastern Siberia (Additional file 1: Fig. S1). This area is situated in an intermontane depression containing wide river valleys, maritime plains and seacoasts [31]. The study sites were selected to access the young through the oldest epigenetic (frozen after sedimentation) permafrost of freshwater and maritime origin [4]. The first site (AL1_15) was located on the right bank of the Alazeya River (69º20.438'N, 154º59.713'E); the second site (CH1_17) was located at a distance of ~ 225 km north-east on the Chukochy Cape at the East Siberian Sea coast (70°04.903'N, 159°55.282'E). The drilling sites were selected to access epigenetic permafrost deposits with known geology and different salinity. The age of the epigenetic permafrost in study sites was estimated based on climatic trends and indirect proofs, e.g., mammalian fauna fossil records or pollen analyses, as described earlier [4, 32]. The permafrost cores (25.8 m from borehole AL1_15 and 21.7 m from borehole CH1_17) were unearthed in August 2015 [33] and August 2017 [34], respectively, using UKB 12/25 drilling rig (Machine-Building Plant named after V.V. Vorovsky, Yekaterinburg) that operates without any drilling fluids. One borehole was drilled at each site. Drilling a single borehole is a standard operational practice for deep terrestrial and ocean drilling [35], which is structured within the project planning, time management and cost estimates. Core samples were collected following a protocol for aseptic sampling described previously [17]. Briefly, the outside layer of extracted cores was trimmed away with alcohol sterilized knife and the permafrost core kernels with diameter of ~ 5 cm were placed into sterile Whirl–Pak® sample bags and stored at − 20 °C until analyses. Cores at each 10 cm depth intervals were subsampled for geochemical and microbiological analysis. Temperature inside boreholes was measured at different depths with the HOBO U12-008 data loggers using Air/Water/Soil Temperature Sensors (Onset Computer Corporation, Bourne, MA).

### Sample description and geochemical analysis

Samples collected from borehole AL1_15 at depths of 2.95, 6.0, 15.0 and 25.1 m below the surface represented perennially frozen alluvial-lacustrine loams and silt loams defined as freshwater permafrost (FP) (Additional file 2: Table S1). The sediments uncovered by borehole CH1_17, based on the physicochemical and micropaleontological analyses, were formed in a coastal zone with a changing water regime [36]. Therefore, the silt loams from the upper horizon (1.75–5.4 m) of borehole CH1_17 were identified to be coastal brackish permafrost (defined as BP) and deeper layers (depths from 11.5 to 19.6 m) were recognized to be saline silt loams and sandy loams of marine permafrost (defined as MP) (Additional file 2: Table S1). The particle size distribution of air-dried permafrost samples was assessed using a standard set of sieves (Science Lab, Ltd.). Detailed geochemical characteristics were described previously [36, 37]. To measure pH (SevenEasy™ pH meter, Mettler Toledo) and conductivity (Ekspert-002 Conductivity meter, Russia), 2.5 g of permafrost was made into a slurry with 5 ml of deionized water. The salinity was calculated from conductivity using the equation described earlier [38]. Oven-dried (105 °C for 24 h) permafrost samples that had been finely ground with a porcelain sterile mortar and pestle were used for total carbon and nitrogen determination at the Stable Isotope Laboratory at the University of Tennessee, Knoxville, United States. Water extract analysis, concentrations of methane and sulfate were obtained at the Soil Cryology Laboratory, Institute of Physicochemical and Biological Problems in Soil Science, Pushchino, Russia. The isotopic signature of methane gas collected inside boreholes was detected using a custom built near-infrared continuous-wave cavity ringdown spectrometer [39] at the Princeton University, Princeton, United States.

### DNA extraction and sequencing

The total community genomic DNA (gDNA) was isolated in duplicate from four (2.95–3.0, 6.0–6.1, 15.0–15.1 and 25.1–25.2 m) core samples of borehole AL1_15 and five (1.75–1.8, 5.4–5.45, 11.5–11.6, 16.9–17.0 and 19.6–19.7 m) core samples of borehole CH1_17 (hereafter, the samples will be referred by upper depth). To overcome limitations connected to a single borehole per site, we instigated a multi-replicate sample analysis. Therefore, intact frozen cores were cut in two pieces and each piece was subsampled for gDNA extraction. The gDNA was extracted from ~ 0.5 g of soil using FastDNA Spin Kit for Soil according to the manufacturer’s instructions (MP Biomedicals). Due to the low biomass, eight to sixteen extractions were pooled for each replicate sample. The pooled gDNA from each sample was concentrated using 100% ethanol (2–2.5 times of the pooled DNA volume) and 5 M NaCl (0.08 times). The mixture was centrifuged at 14,000 g for 25 min at room temperature. The obtained gDNA pellet was resuspended with 35 to 50 µl of DES water (from the FastDNA Spin Kit) and quantified using Qubit 3.0 Fluorometer (Life Technologies). Amount of gDNA varied from 4.8 to 540 ng per 1 g of permafrost.

The V4 region of archaeal and bacterial 16S rRNA genes were amplified using primer set 515F (GTGYCAGCMGCCGCGGTAA) and 806R (GGACTACNVGGGTWTCTAAT) [40]. Samples with higher gDNA concentration were diluted 1:10, and 2.5 μl^−1^ gDNA was taken resulting in 1–5 ng gDNA per 25 μl reaction. After library preparation, the samples were normalized and pooled to a final concentration of 4 pM, combined with 10% PhiX, and loaded on a v3, 600 cycle flow cell, reading 2 × 275 bp paired-end on the Illumina MiSeq. A blank sample was used for sequencing control. Library preparation and sequencing were accomplished at the Genomics Core Facility at University of Tennessee, Knoxville, USA.

The same DNA extracts were used for metagenome libraries preparation using the Nextera DNA Flex Library Preparation Kit and sequenced on Illumina NovaSeq 6000 platform (2 × 150 bp) at Genomics Core Facility at Princeton University, Princeton, USA. The DNA quantity was estimated using an Agilent 2100 Bioanalyzer (Agilent, CA, USA). The input for sequencing was 1–20 ng DNA. The quality metagenomic sequencing data were obtained for four samples of borehole AL1_15 and three samples from borehole CH1_17.

### Bioinformatic analysis

The bioinformatic analyses were carried out using the Infrastructure for Scientific Applications and Advanced Computing resources at the University of Tennessee (project number UTK0011). From amplicon sequencing, a total number of 2.68 million sequences, ranging from 77,598 to 238,405 sequences per sample, were obtained and analyzed using DADA2 v1.10.0 [41]. Briefly, raw reads were filtered and trimmed to remove low quality reads with filtering parameters: truncLen = c(250, 200), maxN = 0, maxEE = c(2, 2), truncQ = 2, and trimLeft = c(19, 20). Trimmed paired-end reads were then merged and used to construct an amplicon sequence variant (ASV) table. ASVs which were found in the sequencing control were removed from the samples using Decontam v1.13.0 [42]. The identified ASVs were annotated using the GTDB database release 89 [43] after chimeric sequences were removed from the ASV table. Taxonomic composition analysis was performed using Phyloseq v1.32.0 [44] and visualized using ggplot2 v3.3.5 [45]. For further analysis, the ASV count was normalized with Phyloseq into percentage as the relative abundance. The alpha diversity indices were plotted using plot_richness function in Phyloseq by choosing measures of “Shannon (H)” and “Simpson (D)”.

Metagenome analysis was carried out first by checking qualities of the raw reads using fastqc. Adapters and low-quality reads were then removed with Trimmomatic v0.36 using the following parameters: ILLUMINACLIP:NexteraPE-PE.fa:2:30:10 LEADING:3 TRAILING:3 SLIDINGWINDOW:4:15 MINLEN:36 [46]. Trimmed reads from all the metagenomes were co-assembled into contigs using Megahit v1.2.9 [47] with a kmer list of 31, 41, 51, 61, 71 and 81. The trimmed reads from each sample were then mapped back to the co-assembled contigs using bowtie2 v2.2.9 [48], samtools v1.3.1 [49] and Picard Markduplicates [50] with default parameters. Binning utilized the Metabat v2.12.1 [51] with contig length cutoff of 1500 bp to generate near complete genomes. The quality of all the generated bins was evaluated using CheckM [52]. Bins with a completeness ≥ 50% and contamination ≤ 10% were selected (defined as metagenome assembled genomes (MAGs)). Taxonomic annotation was performed on selected MAGs using GTDB-Tk v1.0.1 against GTDB database release 89 [43]. Prodigal v2.6.3 [53] was used to predict genes for each selected MAG, followed by annotation with kofamscan v1.2.0 against KEGG database [54]. Coverage for each MAG was obtained using CoverM after removing the 5% of bases with highest and lowest coverages (https://github.com/wwood/CoverM). Relative abundance was calculated by dividing coverage from each MAG by the total coverage from the corresponding sample. Mean relative abundance was used for samples with technical replicates (Additional file 2: Table S2). A network analysis between MAGs and the samples were performed using Cytoscape v3.7.2 [55]. A neighbor-joining phylogenetic tree of *cbbL* and *nifH* gene sequences were constructed, respectively, using extracted amino acid sequences and gene reference databases [56, 57]. The sequences were first aligned using MAFFT version 7 [58], and the closely related sequences were visualized using Archaeopteryx v0.9921 [59].

Co-assembled contigs (≥ 1000 bp) were annotated with Kofamscan v1.2.0 against KEGG database [54] after gene prediction with prodigal v2.6.3 [60]. Transcripts per million (TPM) was used as gene abundance and calculated using Salmon v1.0.0 [61]. TPM, which is normally used in RNA sequencing, represents reads per million when it is applied to metagenomes. Heatmaps were generated using R packages ComplexHeatmap v2.5.5 [62] and Pheatmap v1.0.12 [63]. Mean normalized TPM abundance was calculated for samples with technical replicates (Additional file 1: Figures S3 and S5).

## Results and discussion

### Geochemical characteristics

Permafrost in the Alazeya River basin from borehole AL1_15 upper layers (FP 2.95–6.0 m) was formed ~ 10–40 kyr ago, while deeper permafrost sediments (FP 15.0 and 25.1 m) were formed ~ 0.9 and 1.1 Myr ago (Additional file 2: Table S1) [4]. The geological age of permafrost sediments from borehole CH1_17 also increased with depth. The permafrost layer from BP 1.75–5.8 m likely formed ~ 10–100 kyr ago, whereas permafrost from MP 11.5–19.6 m was formed about ~ 105–120 kyr ago (Additional file 2: Table S1) [4, 7]. Temperature in borehole AL1_15 varied from − 1.8 °C in upper layers to − 5.9 °C in deeper layers, whereas temperatures in borehole CH1_17 were lower and decreased with depth from − 4.8 °C in BP sediments to − 8.1 °C in deeper MP layers. Redox potential measured in MP sediments was + 100 – (+ 150) mV [7] and was lower in FP varying from + 40 – (–100) mV in upper layers to − 256 mV in deeper layers [29] indicating anaerobic conditions in permafrost. The temperature and redox characteristics of studied permafrost were comparable with similar deposits from other locations in Kolyma lowland [7, 21, 29, 30].

FP samples from AL1_15 had 0.04–0.11% total dissolved solids and low salinity in the range of 0.1–0.2 ppt (parts per thousand). BP from CH1_17 formed in a coastal zone had 0.09–0.38% total dissolved solids and salinity 0.3–1.3 ppt increasing with depth. The concentration of total dissolved solids and salinity increased to 1.58% and 6.1 ppt, respectively (Additional file 2: Table S1), in the deeper MP layers in the same borehole. Concentration of ions Cl^−^ and Na^+^ showed a similar trend in both boreholes [17, 36], increasing with depth from 7.5 and 0.89 mmol kg^−1^ to 25 and 1.76 mmol kg^−1^, respectively, in freshwater AL1_15 sediments; and from 6.5–55 and 3.7–33.2 mmol kg^−1^ in coastal brackish CH1_17 sediments to 80–230 and 68–193.6 mmol kg^−1^, respectively, in marine permafrost CH1_17 sediments (Additional file 2: Table S1). Concentration of K^+^ was in the range of 0.27–0.66 mmol kg^−1^ in FP; 0.3–0.6 mmol kg^−1^ in BP; and increased from 1.8 to 7.1 mmol kg^−1^ with depth in MP (Additional file 2: Table S1). Ratio of SO_4_^2−^ to Cl^−^ in all samples was below 1 indicating the dominance of Cl^−^ anions in all studied permafrost samples. Cl^−^, Na^+^ and K^+^ are major osmotically active ions. In addition, Cl^−^ is widespread in bacteria and may be involved in the stabilization of membrane potential, regulation of intracellular pH gradients, regulation of key enzymes and salt adaptation [64].

The isotopic signature of methane collected from borehole AL1_15 at a depth of 23 m was − 84.9 ± 0.5‰ for δ^13^C_CH__4_ and -316.2 ± 1.5‰ for δ^2^H_CH__4_ that shows a biogenic origin.

Total carbon in FP ranged from 0.762 to 2.663%; in BP (1.678–2.088%); and in MP (0.214–0.911%). Total nitrogen measured in FP (0.072–0.175%); in BP (0.149–0.158%); in MP at depth of 11.5–16.9 m (0.022–0.077%) and at 19.6 m (0.108%) was lower than total nitrogen in Siberian tundra soils (0.5–2%) [65]. During the previous study of freshwater permafrost samples collected from the Alazeya River site [66], a low concentration of ammonium (< 100 ppm g^−1^ wet soil) and traces of nitrite and nitrate were detected in the upper sediments followed by a layer (12–24 m), where the concentration of ammonium was 3 times as high as that of the top layer. The same study [66] found high concentrations of nitrite (5–110 ppm g^−1^ wet soil), nitrate (65–115 ppm g^−1^ wet soil) and ammonium (110–152 ppm g^−1^ wet soil) in the Chukochy Cape marine sediments formed 100–150 kyr ago, while only ammonium was detected in brackish sediments in concentration of 22–85 ppm g^−1^ wet soil.

### Microbial diversity from 16S rRNA gene sequencing

**Fig. 1:**
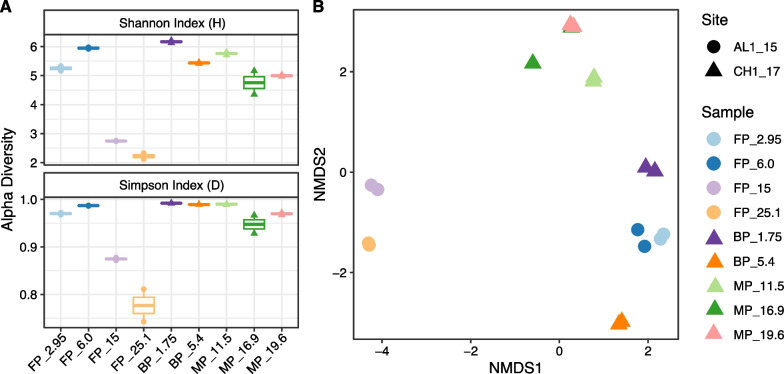
Alpha diversity measured by Shannon and Simpson indexes (**A**) and NMDS ordination plot (**B**) based on 16S rRNA gene amplicon sequencing for two replicates. Unique shape was assigned to borehole AL1_15 (circle) and borehole CH1_17 (triangle). Samples from different depths were distinguished by color. FP—freshwater permafrost; BP—brackish permafrost; MP—marine permafrost; following number indicates depth

In the freshwater permafrost from AL1_15, alpha-diversity determined by the Shannon and Simpson indexes was higher in the shallow and younger permafrost samples and biodiversity generally decreased with depth and age (Fig. 1A). A similar trend was observed in CH1_17 (Fig. 1A).

**Fig. 2:**
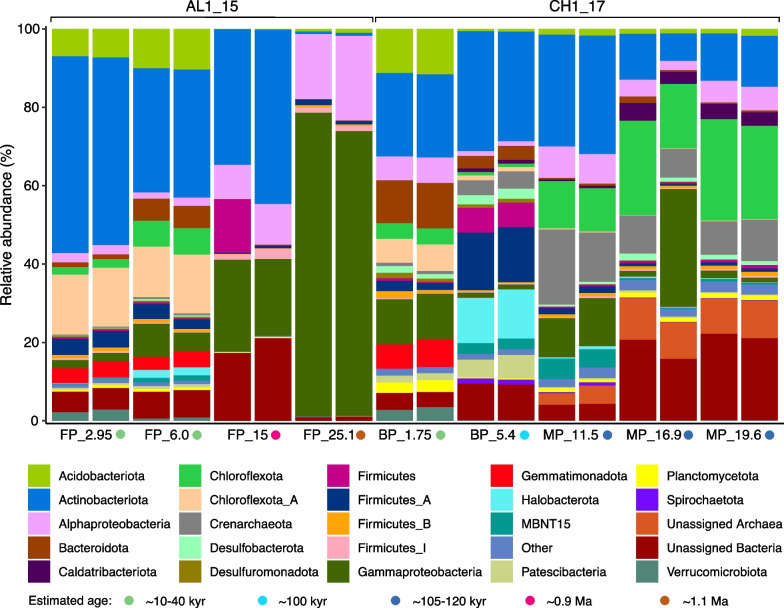
Microbial community compositions at the phylum level (class level for Proteobacteria) determined by 16S rRNA amplicon sequencing. Phyla with abundance < 1% were grouped into ‘Other’. Two replicates were done for each sample

Previous studies of freshwater Siberian [67, 68], slightly acidic high Arctic Canadian [69] and Alaskan tundra and boreal forest [70] permafrost and active layer sediments showed a decrease in biodiversity with increasing of depth, however the latter study found that depth-wise abundance pattern varied by sites [70]. The decrease in diversity with age is in accordance with earlier findings in ancient permafrost [17, 71]. Notably, despite the similar origin and close estimated age, biodiversity in MP_11.5 was higher than in MP_16.9 and MP_19.6 (Fig. 1A), which may be related to conditions of sedimentation or small-scale heterogeneity connected to sample depth. Prior studies of marine permafrost revealed existence of unfrozen saline (170–300 g l^−1^) zones with higher microbial cell concentration and diversity in comparison to surrounding frozen deposits [7, 21]. The nonmetric multidimensional scaling (NMDS) analysis of all amplicon sequence variants (ASVs, based on relative abundance) showed that the microbial communities were different depending on age and salinity (Fig. 1B). In both boreholes the younger samples from upper 6.1 m depths grouped separately from deeper and older permafrost. Despite having different salinities ranging from 0.1 ppt in young FP to 0.3–1.3 ppt in BP, the FP that formed ~ 10–40 kyr ago and BP that formed ~ 10–40 and 100 kyr grouped in close proximity to each other, separately from deeper, older, and more saline permafrost. Based on 16S rRNA gene relative amplicon abundance from duplicate extractions, Actinobacteriota was the most abundant phylum at 2.95, 6.0 and 15 m in FP, while microbial communities from 25.1 m were dominated by Gammaproteobacteria (Fig. 2).

The increase of Gammaproteobacteria in the deeper FP samples (15 and 25.1 m) corresponded to an increase in salinity, total carbon, total dissolved solids, and silt concentration (Additional file 2: Table S1). The Gammaproteobacteria increase could be explained by their ability to adapt to cold conditions, produce low temperature-adaptive enzymes, and maintain cell integrity at temperatures down to − 10 °C [72–74]. Previous studies of freshwater and saline lakes and ponds showed a similar trend when Actinobacteria were more abundant in freshwater and low salinity waters [75] while Gammaproteobacteria increased with increasing salinity [75, 76]. There was no correlation between abundance of Gammaproteobacteria and salinity in samples from CH1_17. Acidobacteria were discovered in all samples except FP_15, with higher representation in low-salinity samples FP_2.95, FP_6.0 m (6.49 and 8.7%, respectively) and BP_1.75 (8.5%). Acidobacteria were only found in the younger upper sections of the cores, perhaps suggesting they are ill-suited to the extreme long-term exposure to permafrost over hundreds of thousands of years.

In CH1_17, the low-salinity top permafrost layers (1.75, 5.4 m) and 11.5 m were dominated by Actinobacteriota (21.3, 29.3, and 29.4%, respectively) and shifted to Chloroflexota in MP at 16.9 and 19.6 m (20.3 and 24.9%, respectively), 11.5 m sample contained representatives of both Chloroflexota (11.7%) and Actinobacteriota (29.4%) phyla. Members of the Chloroflexi phylum are dominant in numerous subseafloor environments [77] that is consistent with our finding of high amounts of Chloroflexota in marine permafrost. ASVs from spore-forming Clostridia (Firmicutes_A phylum) and Bacilli (Firmicutes phylum) classes were, on average, most abundant at 15.0 m in FP (7.41%). These ASVs increased from a mean relative abundance of 2.74% at 1.75 m to 20.69% at 5.4 m in BP, and then declined to 2.10, 1.18 and 1.57% at 11.5, 16.9 and 19.6 m, respectively, in MP. The highest abundance of the Firmicutes was observed in BP at 5.4 m with moderate salinity of 1.3 ppt (1.1 mS cm^−1^) and methane concentration of 76 µmol kg^−1^. Previous studies showed a positive correlation between relative abundances of Firmicutes and either salinity gradient of 0.36–6.72 mS cm^−1^ in temperate soils [78] or CH_4_ production in anaerobic digester [79]. Archaea were detected in all the samples except for FP_25.1. Halobacterota and Crenarchaeota contributed a relatively high abundance in BP at 5.4 m and in MP at 11.5 m (averaging 16.32 and 16.46%), respectively (Fig. 2). Within the Halobacterota, ASVs associated with methanogens, such as *Methanobacteria*, *Methanosarcinia*, *Methanomicrobia*, *Syntrophoarchaeia* and *Methanocellia*, were most abundant at 6.0 m in FP (averaging 2.09%) and at 5.4 m in BP (averaging 8.52%). While these samples originated in lake and coastal mixing zones, respectively, and were collected at similar depths, the sample from 5.4 m had 10 times higher salinity, and more than twice the abundance of carbon, nitrogen, and methane (Additional file 2: Table S1). The presence of methane at 5.4 m is supported by presence of salt-tolerant methanogens, such as *Methanobacteria*, *Methanocellia*, *Methanomicrobia* [80]. Decreased abundance of the methanogens in deeper MP samples may be connected to the increase of SO_4_^2−^. Similar to our study, negative correlation between presence of SO_4_^2−^ and CH_4_ production in artificial sea water microcosms was reported [25]. Methanogenic archaea were not identified at 15.0 and 25.1 m in FP despite a high concentration of methane in those samples. This methane could therefore be explained by methane compression in deeper layers during epigenetic freezing from the top down [81].

### Identification and distribution of MAGs

**Fig. 3:**
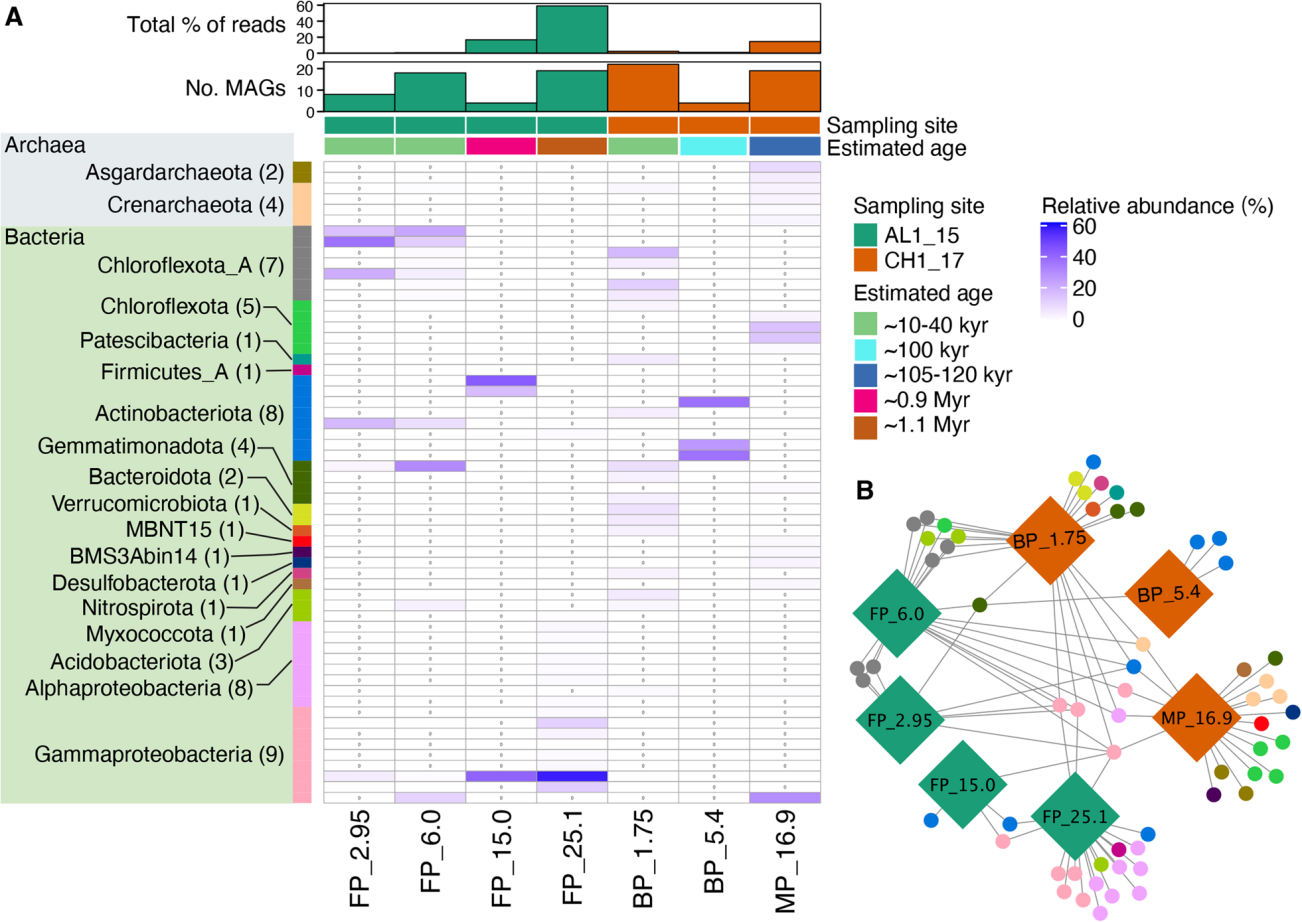
Distribution of the recovered MAGs and their relative abundance. (**A**) Heatmap showing relative abundance of the recovered MAGs. The bar plots show the total percentage of sequencing reads mapped to the MAGs and the total number of MAGs recovered in the corresponding sample. Numbers given in brackets indicate the number of MAGs assigned to the corresponding taxon. The uncolored heatmap cells with 0 written in them indicate the absence of that MAG. (**B**) Network showing the number of MAGs recovered from each sample of the two sampling sites. Each MAG is presented by circle, which are color coded for the suggested taxonomy shown in A

Metagenomic binning from the co-assembly of the seven permafrost samples (two marine permafrost samples failed to yield metagenomes of good quality) resulted in a total of 60 MAGs with ≥ 50% completeness and ≤ 10% contamination, altogether contributing 0.24 to 59.10% of sequencing reads of the respective samples, with the highest proportion of reads in FP_25.1 and lowest in FP_2.95 (Fig. 3A). The low percentage of reads recovered in MAGs from young permafrost samples may be attributed to higher biodiversity in that sample (Fig. 1A). Because of different coverages of metagenomes, the co-assembly provided obvious benefits for capturing more of the diversity due to higher read depth, robust assembly, improved MAGs recovery [82], and facilitated comparison across permafrost samples. The reconstructed MAGs comprised phylogenetically diverse members from 2 archaeal and 15 bacterial phyla (Fig. 3A and Additional file 2: Table S2). Of the 60 recovered MAGs, 15 MAGs were retrieved from both boreholes, while 20 MAGs were only identified in AL1_15 and 25 MAGs were exclusively found in CH1_17. Clearly partitioned communities were observed between the two boreholes (Fig. 3B). Network analysis showed that deepest freshwater (FP_25.1) and marine (MP_16.9) permafrost samples had high number of unique MAGs, 14 MAGs in each sample, followed by 8 and 3 unique MAGs in brackish (BP_1.75 and BP_5.4) permafrost samples, respectively (Fig. 3B). No sample-unique MAGs were discovered in freshwater young samples (FP_2.95 and FP_6.0). MAGs aligning with Asgardarchaeota, Bacteroidota, BMS3Abin14, Desulfobacterota, MBNT15, Myxococcota, Nitrospirota, Patescibacteria and Verrucomicrobiota were only identified in CH1_17, while members of phylum Firmicutes_A were exclusively detected in AL1_15.

Microbial community composition and metabolic traits varied with sediment depth and permafrost age. In CH1_17, the number of recovered MAGs was highest in BP_1.75 (n = 22) and lowest in BP_5.4 (n = 4). The majority of the recovered populations from BP were related to Chloroflexota_A at 1.75 m (40.53%, all quoted percentage values in the sections below refer to the relative abundance from the respective metagenome) and Actinobacteriota at 5.4 m (99.96%), whereas the recovered populations from MP at 16.9 m were mostly related to Chloroflexota and Gammaproteobacteria (34.93 and 29.21%, respectively). In addition, Acidobacteriota, Bacteroidota, Chloroflexota_A, Nitrospirota, Patescibacteria and Verrucomicrobiota MAGs were exclusively observed at 1.75 m, while BMS3Abin14, Desulfobacterota, MBNT15 and Myxococcota were only detected at 16.9 m. Lastly, archaeal MAGs aligning with Asgardarchaeota, which is suggested to be the closest prokaryotic relatives to eukaryotes [83, 84], were also only recovered from MP at 16.9 m. Another analysis of MAGs obtained from discrete depths of marine permafrost and annotated against TIGRFAM and COG [37] showed the presence of Asgardarchaeota, Bacteroidetes, Nitrospirae, and Deltaproteobacteria. Contemporary descendants of the microorganisms discovered in CH1_17, e.g., Chloroflexota, candidate lineage MBNT15, sulfate-reducing bacteria, were found to be highly metabolically flexible organisms that could adapt to resource variability by using different electron donors and acceptors or being involved in syntrophic interactions [85–87]. For example, Myxococcota from anoxic aquatic environments being a strict anaerobe are capable of using fermentation, nitrate reduction, and dissimilarity sulfate reduction for energy acquisition [88] which is in agreement with the genes found in our MAGs. The current discovery of MAGs for Desulfobacterota and MBNT15 in deeper MP_16.9 sample (30.31 mmol SO_4_^2−^ kg^−1^) are in line with the previously obtained data that obligately anaerobic taxa such as sulfate reducers and candidate lineage MBNT15 thrive in more stable deeper marine sulfate-rich sediments [87]. Nevertheless, ASVs from Desulfobacterota were identified in all samples at varying concentration of sulfate (1.61–36.84 mmol kg^−1^) except oldest sample FP_25.1 with lowest sulfate (0.64 mmol kg^−1^). In absence of sulfate, the sulfate reducing bacteria are able to ferment organic acids and alcohols, producing hydrogen, acetate, and carbon dioxide [85]. Having different metabolic capabilities raises the chance of survival in environments when electron acceptors become depleted. The 2 MAGs belonging to Asgardarchaeota were also discovered in the deeper marine sample. A recent review suggested that many Asgardarchaeaota are involved in syntrophic interactions [89], e.g., the syntrophic exchange of formate and hydrogen was shown between a Lokiarchaeon and a sulfate-reducing Deltaproteobacterium [90].

In freshwater permafrost from AL1_15, the metagenome from 25.1 m generated the highest number of MAGs (n = 19), followed by 6.0, 2.95 and 15.0 m (n = 18, 8 and 4, respectively). Even though the FP_25.1 metagenome size was approximately half that of the FP_2.95 metagenome, it generated the highest number of MAGs. This could be explained by low biodiversity in sample FP_25.1 based on the 16S rDNA amplicon analysis (Fig. 1A). While poor MAG recovery from younger permafrost samples could be related to high diversity of the microbial community [91]. A high abundance of the recovered populations was aligned with Chloroflexota_A at 2.95 and 6.0 m (73.73 and 44.27%, respectively), whereas MAGs recovered from 15.0 and 25.1 m were dominated by Actinobacteriota (59.16%) and Gammaproteobacteria (89.57%), respectively. In addition, populations affiliated with Chloroflexota_A and Gemmatimonadota were only identified at 2.95 and 6.0 m, while Firmicutes_A MAGs were exclusively detected at 25.1 m.

Amplicon and metagenome approaches detected similar populations of bacteria. However, amplicon analysis showed presence of some bacteria in more samples than was detected by metagenomics. The number of MAGs correlated positively with microbial diversity based on amplicon analyses in BP samples (higher number of MAGs in BP_1.75 at higher Alpha diversity), and correlated negatively in FP samples (higher number of MAGs in FP_25.1 at lower Alpha diversity). This discrepancy may be connected to biases during isolation of DNA from samples with different salinity, sequencing approaches, depth of sequencing, platform for sequencing technology and bioinformatic approaches [92].

### Metabolic potential in permafrost

**Fig. 4:**
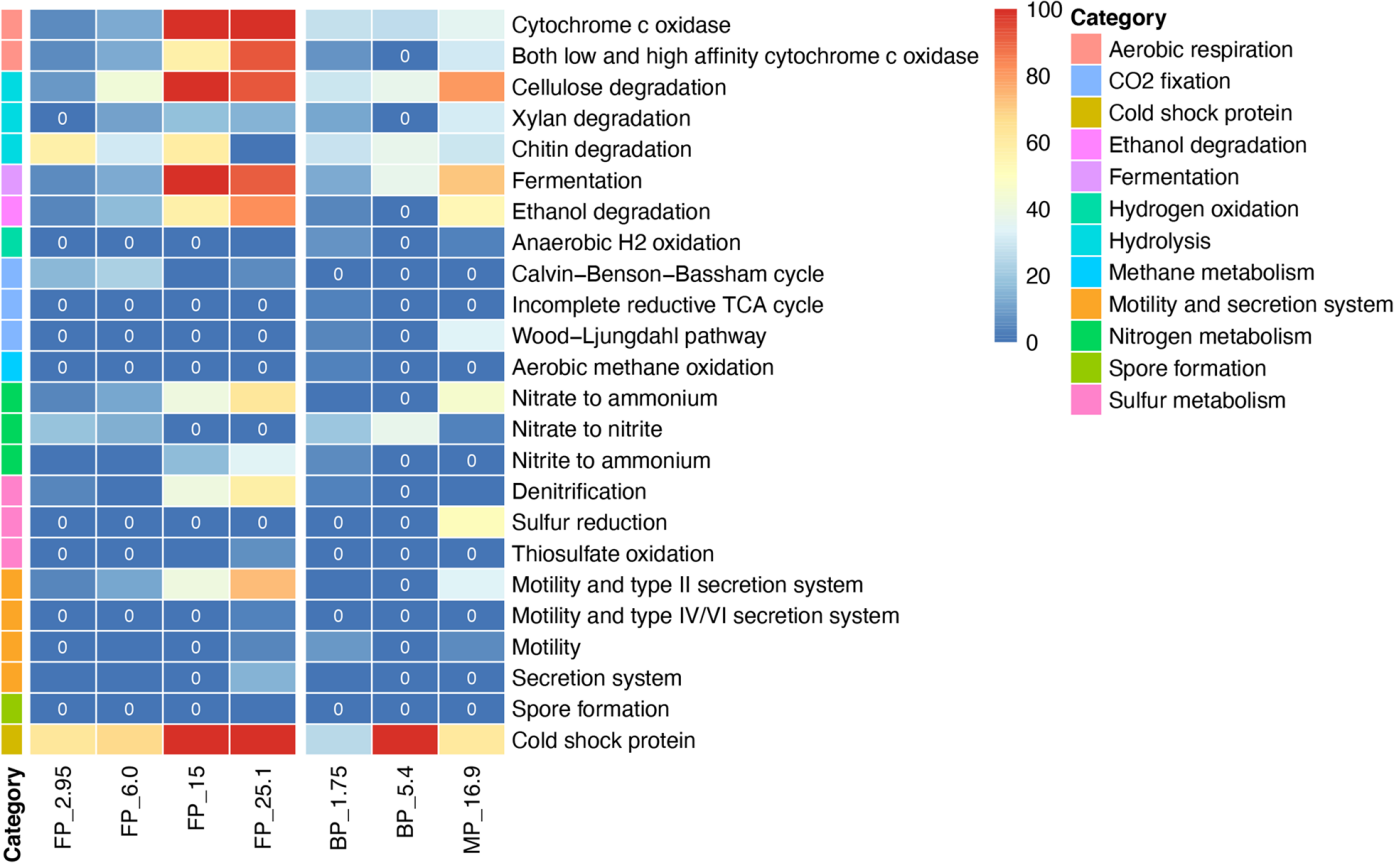
Heatmap showing the total relative abundance of MAGs containing genes encoding the corresponding metabolic function from the respective samples. The 0 written in the dark blue heatmap cells indicates the absence of that gene

**Fig. 5:**
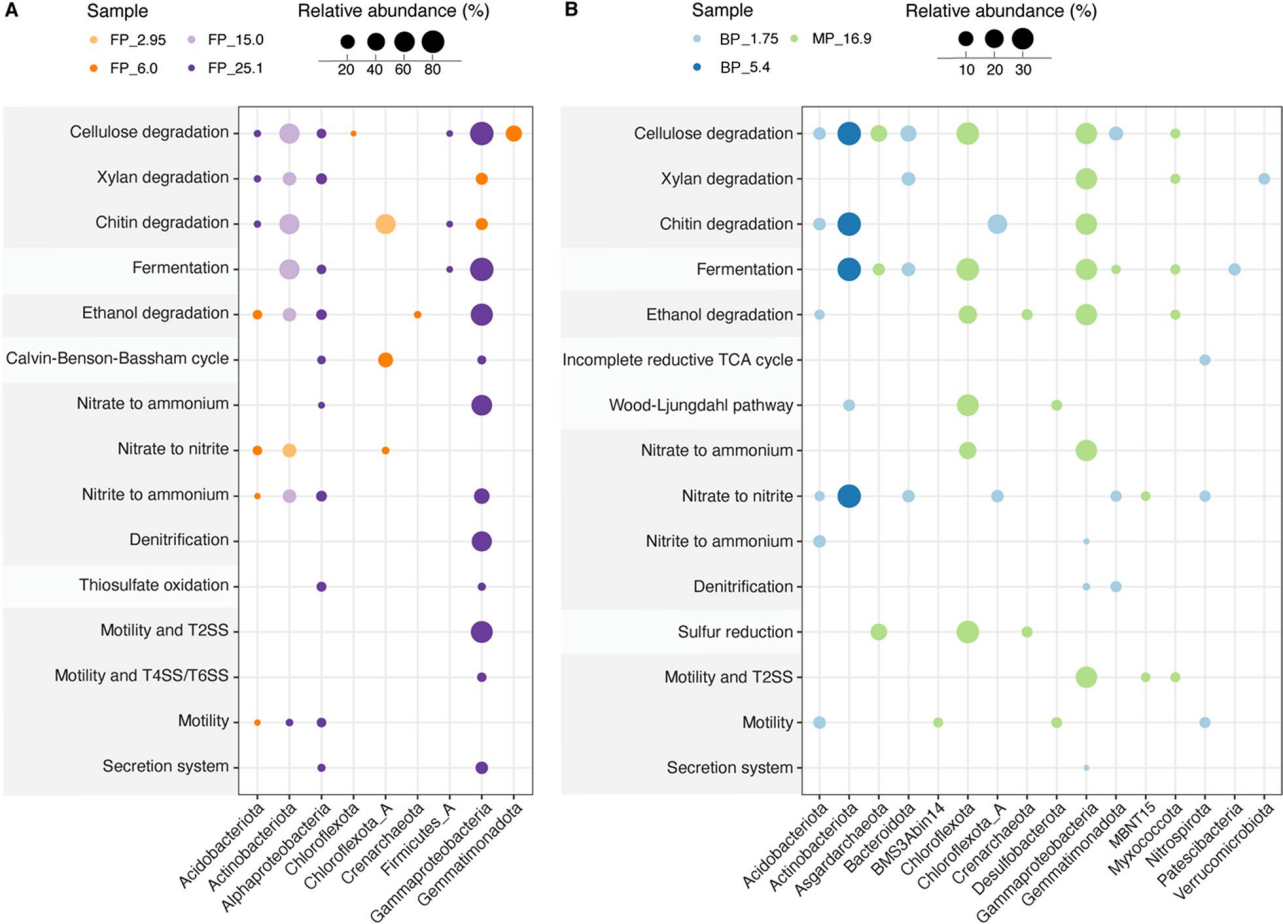
Genome-enabled metabolic potential per taxonomic distribution of freshwater (**A**) and brackish and marine permafrost (**B**). The plots show the total relative abundance of different function and taxon from the respective metagenomes. Only the sample with highest relative abundance is shown for each function and taxon. Full taxonomic ranges of all described functions are presented in Additional file 2: Table S3

**Fig. 6:**
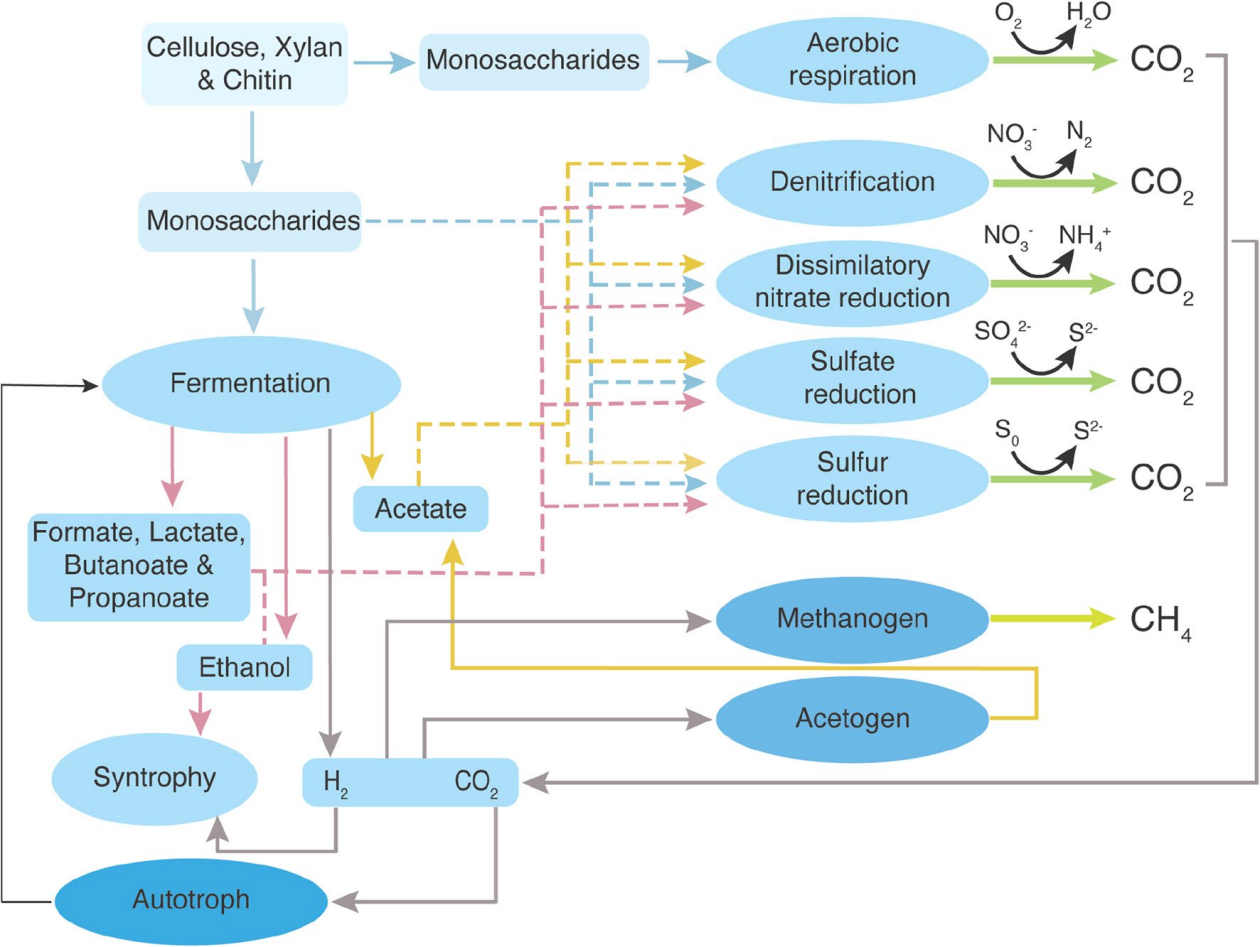
Flow diagram of the potential metabolic pathways derived from gene assignments from the MAGs reconstructed in this study and the un-binned metagenome data. Arrows represent metabolic capabilities that were identified in this study. The dashed lines represent potential electron donors for the anaerobic respiration processes

Recovered MAGs were screened for genes encoding hydrolysis, fermentation, respiration, CO_2_ and N_2_ fixation, motility, bacterial secretion, spore formation and stress resistance (Figs. 4, 5 and Additional file 2: Tables S3–S5) to identify their metabolic potential. Genes coding for certain functions that were not identified in MAGs or in many samples were also searched in un-binned, assembled metagenomic sequences and a summary of potential metabolic pathways identified in permafrost metagenomes presented in Fig. 6.

#### Aerobic respiration

Despite the scarcity of oxygen (< 0.07 mg O_2_ kg^−1^ at redox potential below + 200 mV [93]), genes encoding the machinery to reduce oxygen were identified in 30 MAGs, including members of Acidobacteriota, Actinobacteriota, Alphaproteobacteria, Bacteroidota, Chloroflexota, Gammaproteobacteria, Gemmatimonadota, MBNT15 and Nitrospirota. Depending on the sample, 5.29–100% of the recovered populations contained genes coding for cytochrome *c* oxidase (Fig. 4).

These communities were most abundant at 15.0 and 25.1 m in FP (100 and 99.76%, respectively) and at 16.9 m in MP, respectively (35.69%). The high abundance of genes coding for cytochrome *c* oxidases could be explained by high identity of this enzyme in diverse organisms [94]. Cytochrome *c* oxidase catalyzes redox-driven proton pump that take part in generating the proton gradient in both prokaryotes and mitochondria that drives synthesis of ATP [94]. This highlighted the importance of energy acquisition through oxygen reduction in the permafrost populations, and accumulation of these enzymes in the deeper and older samples. In addition, MAGs containing genes encoding both low and high affinity cytochrome *c* oxidase had the highest relative abundance at 25.1 m in FP (92.49%) and at 16.9 m in MP (30.94%, Fig. 4), suggesting that the recovered populations from the deeper samples were capable of operating under different levels of O_2_ concentration. This is in line with previous cultivation studies showing that isolates from the Siberian permafrost grew well at atmospheric oxygen concentrations and at NaCl up to 10% [12, 30, 95].

#### Carbohydrate hydrolysis

Chitin and plant-derived materials, such as cellulose and xylan, are the two most abundant types of polysaccharides in the ecosystem [96]. MAGs containing genes encoding cellulases and/or β-glucosidases, which are involved in cellulose degradation, were most abundant in ancient (0.9–1.1 M old) freshwater permafrost at 15.0 and 25.1 m depth (100 and 92.63%, respectively) and in 120 K old marine permafrost at 16.9 m (80.85%) (Fig. 4) with carbon content of 1.896, 2.663 and 0.911%, respectively. The populations in samples with contrasting salinity dominated by different bacteria which are Actinobacteriota in FP_15.0, Gammaproteobacteria in FP_25.1, and Chloroflexota and Gammaproteobacteria in MP_16.9 (Fig. 5).

MAGs with the capacity of carrying out xylan degradation were identified in all the samples irrespectively of the salinity besides FP_2.95 and BP_5.4. Approximately 10.99–31.31% of the recovered communities contained xylanase and/or β-xylosidase genes, with the highest at 15.0 m in FP (17.29%) and 16.9 m in MP (31.31%), where these potentially xylan degrading microorganisms were dominated by Actinobacteriota and Gammaproteobacteria, respectively. The higher abundance for cellulases over the protein-coding sequences for xylan breakdown shows that the Siberian permafrost populations have potential to degrade cellulose, the most commonly utilized polysaccharide. A recent study of Alaskan permafrost with ages of 19–33 kyr showed that sequences for enzymes targeting structural polysaccharides, such as xylan and cellulose, were less abundant than those targeting smaller molecular weight compounds [97]. However, the same study displayed that microbial community changed along the chronosequence from young permafrost community with potential to target hemicellulose, through increased potential to target starch, to the old permafrost microbial population having enzymes to target recalcitrant substrates like peptidoglycan and cellulose, that is in line with our findings. Genes predicted to encode enzymes of chitin degradation pathway were most abundant at 2.95 and 15.0 m in FP (57.82 and 59.16%) and at 5.4 m in BP (37.21%). Both MAG_99 and MAG_142 affiliated with Actinobacteriota were the dominating population for chitin degradation at 15.0 m in FP and at 5.4 m in BP, respectively, whereas the dominant chitin degrading population at 2.95 m in FP belonged to one Chloroflexota_A MAG.

#### Fermentation

The potential for fermentation was identified in 23 MAGs, including members of an archaeal phylum, Asgardarchaeota. Microorganisms with the capability of producing acetate and/or lactate via fermentation were identified in all the samples regardless of salinity. In FP sediments, abundance of fermentation-encoding microorganisms increased with depth and contributed 100 and 91.76% of the recovered communities at 15.0 and 25.1 m, respectively, while approximately 5.29 and 12.02% of the recovered populations contained genes for fermentation at 2.95 and 6.0 m, respectively (Fig. 4). The most abundant MAGs encoding fermentation pathways were Gammaproteobacteria at 2.95, 6.0 and 25.1 m, whereas these populations primarily belonged to Actinobacteriota at 15.0 m (Fig. 5). Formate fermentation genes were only observed in ~ 40 kyr old freshwater permafrost at 6.0 m, whereas butanoate fermentation genes were exclusively identified in the oldest ~ 1.1 Myr sediments at 25.1 m. The recent metagenomics study of the alluvial silty loams continuously frozen for 0.01–1.1 Myr showed that genes involved in the synthesis of formate, acetate, and butyrate were more numerically abundant in the older sediments [17]. In addition, MAGs containing genes indicating the capability for fermentative production of ethanol were identified at 6.0 and 25.1 m in FP and at 16.9 m in MP. In BP and MP, genes encoding fermentation were most enriched in MP_16.9 (71.46%), followed by BP_5.4 and BP_1.75 (37.21 and 12.79%, respectively). Chloroflexota and Gammaproteobacteria MAGs appeared to be the most abundant groups with pyruvate metabolic genes at 16.9 m, while the most abundant fermentation encoding microorganisms were Bacteroidota at 1.75 m and Actinobacteriota at 5.4 m. Populations containing genes for formate and butanoate fermentation pathways were identified at 1.75 m in BP and 16.9 m in MP. Our results are in line with analyses of neighboring samples that showed presence of low–molecular-weight organic acids, such as formate, acetate, and propionate, at all depths with the highest concentrations of acetate (36.2 ± 3.3 μg g^−1^) and formate (12.0 ± 0.7 μg g^−1^) in BP at depth of 5.8 m [37]. Lastly, one Myxococcota MAG containing genes encoding pyruvate fermentation to propanoate was exclusively identified in MP at 16.9 m. Overall, our data suggested that fermentation contributed significantly to anaerobic degradation of soil organic carbon in the deeper and older permafrost (Fig. 4 and Additional file 2: Table S4) independently from salinity, which is in line with previous studies emphasizing its importance in permafrost [69, 98].

#### Potential metabolic interaction between populations

Short-chain fatty acids, alcohols, hydrogen and CO_2_ converted by fermentative populations can subsequently be used by other microorganisms. Genes involved in ethanol degradation were detected in all the samples except for BP_5.4. They were particularly abundant at 25.1 m in FP (82.63%) and at 16.9 m in MP (53.68%, Fig. 4). These populations were dominated by Gammaproteobacteria in FP_25.1 and MP_16.9 (Fig. 5). Hydrogen is an important electron donor for microorganisms in anaerobic conditions. MAGs containing homologs for anaerobic hydrogen oxidation were only identified in FP_25.1, BP_1.75 and MP_16.9 (0.24, 7.43 and 3.15%, respectively). These populations belonged to Bacteroidota (n = 1), Desulfobacterota (n = 1) and Firmicutes_A (n = 1). However, the NADP-dependent hydrogenase catalyzes reversible oxidation of H_2_, therefore, the detected hydrogenase could also be involved in hydrogen production.

Five MAGs containing genes coding for form I ribulose-bisphosphate carboxylase (RuBisCO) were only identified in FP (Additional file 1: Fig. S2 and Additional file 2: Table S2). The form I RuBisCO is the key enzyme in CO_2_ fixation via the Calvin-Benson-Bassham (CBB) cycle and the most abundant form in bacteria and eukaryotes [99]. Two Alphaproteobacteria MAGs and two Gammaproteobacteria MAGs had both *cbbL* and *cbbS* genes for form I RuBisCO, while the Chloroflexota_A MAG only contained the *cbbL* gene. These microorganisms were most abundant in FP_6.0 (22.84%) and were mainly presented by Chloroflexota_A (Figs. 4 and 5). In addition, three archaeal MAGs, from Asgardarchaeota and Crenarchaeota, were suggested to hold *cbbL* genes encoding form III RuBisCO and were only identified in MP at 16.9 m (10.59%). The form III RuBisCO is known to be present in many archaea [57] and reported to be involved in the incorporation of CO_2_ into ribulose-1,5-bisphosphate (RuBP) from nucleotides like adenosine monophosphate (AMP) [100, 101]. Genes coding for form IV RuBisCO were identified in 3 MAGs, including the two MAGs also containing genes for form I and one archaeal MAG also with genes for form III. Notably, form IV, which is often referred to as a RuBisCO-like protein, appears to be involved in sulfur metabolism, methionine salvage pathway, and D-apiose catabolism [102, 103], rather than the CBB cycle. The microorganisms carrying this form were found in FP at 2.95, 6.0 and 25.1 m and in MP at 16.9 m. MAG_40, closely related to Nitrospirota, encoded genes assigned to CO_2_ fixation via incomplete reductive tricarboxylic acid (TCA) cycle, and was exclusively identified in BP at 1.75 m (3.38%). Lastly, key genes encoding both anaerobic carbon-monoxide dehydrogenase and acetyl-CoA synthase for the Wood-Ljungdahl pathway were found in 4 MAGs. The Actinobacteriota MAG was only identified in BP at 1.75 m (4.19%), whereas the Desulfobacterota and Chloroflexota MAGs were detected in MP at 16.9 m (33.64%). Overall, the higher abundance of CO_2_ fixation potential in MP at 16.9 m (33.64%) suggested that autotrophy was more widespread in saline marine environment when this permafrost layer formed relatively to the others.

#### Methane metabolism

Methane is a potential electron donor for anaerobic respiration, coupled to sulfate, iron, manganese, nitrate, nitrite reduction or denitrification [104–110]. It is also a potent greenhouse gas that contributes to global warming. The recovered MAGs as well as un-binned metagenomic sequences were screened for genes encoding for methanogenesis, anaerobic and aerobic methane oxidation. None of the MAGs were suggested to carry genes involved in anaerobic methane oxidation (AOM) or methanogenesis. However, within the un-binned metagenome data, the key genes involved in methanogenesis (based upon the presence of the *mcrBCDG* genes) were identified in all samples except FP_15.0 and BP_1.75 (Additional file 1: Fig. S3). These genes were most enriched in BP_5.4 (367.46 TPM), corresponding to the 16S rRNA amplicon data showing the highest relative abundance of methanogenic taxa, and correlated with a presence of methane in layer between 4.8 and 6.4 m with the highest concentration of methane 76 µmol kg^−1^ detected near this depth at 5.6 m. The concurrent presence of genes involved in methanogenesis, ASVs associated with methanogens and methane detected by the static headspace method [81] suggests that methane at 5.6 m depth has a biological origin and methane production may happen in situ at brackish permafrost conditions. Overall, the capacity for methanogenesis appeared to be relatively low in the remaining samples (0.006–7.81 TPM) which is in line with the previous studies showing that methanogenesis was extremely limited in intact permafrost [19, 111]. Despite high levels of methane 157.1 and 167.2 µmol kg^−1^ detected in FP at 15.0 and 25.1 m, respectively [81], the key genes for methanogenesis were not identified at 15.0 m and were negligible at 25.1 m (0.006 TPM). A previous study, which sampled permafrost from the same location at three depths (1.4, 11.8, and 24.8 m), also showed low abundance of methanogens [17]. The low abundance of methanogenesis genes in samples with high concentrations of methane that has biogenic origin (− 84.9 ± 0.5‰ for δ^13^C_CH__4_) may suggest that this methane has either surficial or deep-sediment origin and likely accumulated in lithological traps earlier during permafrost formation [81] or represents a consequence of methanogenesis in deeper permafrost layers, similar to observations from organic-rich Antarctic marine sediments where the methanogenesis genes are in low abundance despite the observation of biogenic methane [112]. Low abundance of methanogens in metagenomes from permafrost samples with biogenic methane may also be attributed to biases associated with sample size, DNA extraction, sequencing technology, depth of sequencing, sequence assembly, annotation, and database used for identification [92]. Analysis of replicate metagenomes and ultra-deep sequencing of additional samples from methane containing permafrost layers would likely result in more prominent detection of methanogens.

In contrast to the rare occurrence of anaerobic methane oxidation in permafrost, aerobic methane oxidation typically attenuates methane release [113, 114]. However, MAG_40, encoding the capacity to oxidize methane aerobically and most closely related to Nitrospirota, was only found in BP at 1.75 m at abundance 3.38% (Fig. 4). Within the un-binned metagenomic sequences, genes involved in aerobic methane oxidation (based on the presence of the *pmoABC-amoABC*) were identified at 2.95 and 6.0 m in FP (1.38 and 4.06 TPM, respectively, Additional file 1: Fig. S3) and at all depths in BP and MP, with highest abundance in BP_1.75 (15.05 TPM). The generally low abundance of genes for aerobic methane oxidation was not surprising considering the lack of oxygen in permafrost. In agreement with the high abundance of methanogenesis genes in BP at 5.4 m, genes for aerobic methane oxidation were lowest at this depth (0.77 TPM), indicating the strictly anaerobic microenvironments at this depth. It should be noted that the identified genes encoding aerobic methane oxidation were also suggested to co-oxidize ammonia aerobically [115].

#### Nitrogen and sulfur metabolism

The potential ability to fix nitrogen was not identified in the recovered MAGs. Within the un-binned metagenome data, nitrogenase genes were identified in all samples regardless of salinity with the exception of FP_15.0. An overall low abundance of form I and III *nifH*, *nifD* and *nifK* genes was observed across the samples (totaling 0.12–16.95 TPM, Additional file 1: Fig. S3 and S4). Genes for the enzymes needed to utilize nitrate or nitrite as a terminal electron acceptor in the process of nitrification were detected in 26 MAGs. Populations that were suggested to reduce nitrate to either nitrite or ammonium as the end products were found in both freshwater and marine sediments and were most abundant in deepest sediments at 25.1 m in FP (62.84%) and at 16.9 m in MP (48.46%). The process of ammonification within the sediments is supported by higher concentration of ammonium in deeper sediments (~ 300 ppm NH_4_^+^ g^−1^ at depth of 12–24 m in FP and 110–152 ppm NH_4_^+^ g^−1^ in MP [66]). These populations were dominated by Gammaproteobacteria in both samples. Genes encoding the capacity to reduce nitrite to ammonium were also most enriched at 25.1 m in FP (34.13%) and were identified at 1.75 m in BP with a relatively lower abundance (5.45%). In addition to the dissimilatory nitrate reduction pathway, three MAGs also contained a complete or near complete set of genes coding for denitrification. The denitrifying populations represented a small fraction of the recovered communities, which is present in all the samples (0.03–4.74%), except for deep samples from freshwater permafrost namely FP_15.0 and FP_25.1 (40.84 and 58.19%, respectively). The presence of nitrite and nitrate in different FP layers and in deeper MP layers supports a potential of the communities to the denitrification process. No MAGs were suggested to carry out denitrification in BP_5.4 and nitrate was not detected in BP layer. Overall, our data suggest that nitrogen metabolism plays an important role in the older permafrost, likely due to nitrate being the most energetically favorable electron acceptor in the absence of oxygen. The generally higher abundance of populations involved in nitrate reduction is consistent with previous reports of their dominance under reducing, high-carbon and low-nitrate conditions [116, 117].

Dissimilatory sulfate reduction by anaerobic microorganisms is a predominant pathway of organic material mineralization in marine sediments [118]. However, none of the MAGs contained the key *dsrA* and/or *dsrB* genes for dissimilatory sulfate reduction, suggesting the rare utilization of this pathway by permafrost populations in the studied samples. Populations containing genes encoding enzymes involved in sulfur reduction via polysulfide were exclusively identified in MP at 16.9 m (51.08%). The presence of hydrogen sulfide in MP samples was identified by the specific odor during core extraction in the field. These populations were encoded by Asgardarchaeota, Crenarchaeota and Chloroflexota. Genes encoding thiosulfate oxidation via the Sox pathway were only identified in FP at 15.0 and 25.1 m (0.34 and 6.35%, respectively). Populations containing these genes exclusively belonged to Alpha- and Gammaproteobacteria, where the Alphaproteobacteria MAGs were only identified in FP_25.1. In agreement with the genomic analysis of MAGs, an overall low abundance of the *dsrAB* genes coding for sulfate reduction was detected within the un-binned metagenome data (1.57–18.68 TPM) at 2.95 and 6.0 m in FP, 1.75 and 5.4 m in BP and 16.9 m in MP. Nevertheless, the possibility of the sulfate reduction was supported by the presence of sulfate in FP (4.4–8.8 mmol kg^−1^, except sample FP_25.1 where sulfate was ~ 10 times lower), BP (1.7–2.7 mmol kg^−1^), and MP (8.1–36.8 mmol kg^−1^) samples. The content of sulfate in all studied samples was < 0.2% by mass what is considered low sulfate soils [119]. The previous study also discovered the presence of sulfate reduction in marine and deep freshwater permafrost sediments with S^2−^ in concentration of 0.12–0.22 and 0.12–0.35 g kg^−1^ wet soil, respectively [29]. The phylum Desulfobacterota that comprises sulfate-, sulfur-, and ferric iron-reducing bacteria was solely detected in MP at 16.9 m. In addition, genes encoding the use of polysulfide sulfur were more abundant in MP within the un-binned metagenome data (Additional file 1: Fig. S3), suggesting that anaerobic degradation of organic carbon coupled to sulfur reduction was more common in MP. Of the aforementioned genomes that encoded the capacity for fermentation and/or anaerobic respiration, a high abundance of the recovered communities also contained genes encoding the machinery to reduce oxygen in FP at 15.0 and 25.1 m (100 and 96.96%, respectively). Taking these together, we suggest that the recovered communities from the older samples of FP are likely to be facultatively anaerobes, whereas the recovered populations from 5.4 m in BP to 16.9 m in MP are more likely to be strict anaerobes at the time of freezing (Additional file 2: Table S4).

#### Potential adaptation in permafrost

Biofilm formation is considered to be a survival strategy to enable adaptation of microorganisms to extreme environments [120], i.e., bacteria were tightly associated with soil particles in Siberian permafrost [121]. In addition, *Psychrobacter arcticus* strain 273-4 from Siberian permafrost showed the capability to form biofilms [122]. Genes coding for both surface attachment through flagellar (including chemotaxis) and/or type IV pili and extracellular polymeric substance (EPS) secretion via type II secretion system (T2SS) which are potentially involved in biofilm formation were identified across seven MAGs representing Gammaproteobacteria, MBNT15 and Myxococcota. Biofilm-forming Gammaproteobacteria were most abundant at 25.1 m in FP (73.10%) and decreased in abundance toward the surface (Fig. 4). In BP and MP, the highest abundance of the biofilm-forming microorganisms was detected in MP_16.9 (33.07%). None of the MAGs from BP_5.4 contained genes encoding both surface attachment and EPS secretion. Of the 7 MAGs, one Gammaproteobacteria MAG also included genes for type IV secretion system (T4SS) which encodes conjugation machinery and DNA release and uptake systems [123]. Another three Gammaproteobacteria MAGs, MAG_24, MAG_35 and MAG_67 also contained genes coding for type VI secretion system (T6SS, delivering toxins into eukaryotic and prokaryotic cells [124]), whereas genes encoding type III secretion system (T3SS, injecting effector proteins into eukaryotic cells [125]) were also detected in MAG_35. In addition, three MAGs included genes for flagellar and/or type IV pili assisted motility and T6SS, where one MAG also contained genes encoding T4SS. These microorganisms were exclusively identified in FP at 25.1 m (3.80%). MAGs containing genes for flagellar or type IV pili mediated motility but lacking genes encoding secretion systems were also identified at 6.0 and 25.1 m in FP (0.12 and 4.89%, respectively), 1.75 m in BP (8.81%) and 16.9 m in MP (5.12%). In addition, approximately 0.02–14.24% of the recovered MAGs contained genes assigned to type I secretion system (T1SS), T2SS or T6SS at 2.95, 6.0 and 25.1 m in FP and 1.75 m in BP, with the highest abundance observed in FP_25.1. The high abundance of recovered MAGs that contained genes encoding both surface attachment and bacterial secretion system in FP_25.1 and MP_16.9 indicated that biofilm formation might be an important survival strategy for microbes in the older and deeper perennially frozen sediments, providing exchange of molecules and ions between live microorganisms and liquid brine veins surrounding cells and soil particles [6, 18]. This is generally in agreement with the un-binned metagenome data analysis where genes involved in chemotaxis, flagellar assembly, type IV pili and bacterial secretion systems (type I-III and VI) were more abundant in the oldest permafrost sample (FP_25.1), while genes encoding T4SS were most abundant in MP_16.9 (Additional file 1: Fig. S5). The higher abundance of T4SS in marine permafrost could indicate that horizontal gene transfer may play a role in allowing microorganisms to adapt to changes in their environment. Overall, our data support the findings from a previous metagenome study of Alaska permafrost showing that chemotaxis and bacterial secretion system pathways were enriched in older permafrost up to 33 kyr [71], but also pointed to the presence of bacterial populations that were potentially involved in biofilm formation.

Sporulation is a widely utilized strategy for microorganisms to survive in extreme environmental conditions [126]. MAG_104, most closely related to Firmicutes_A, was suggested to carry out multiple stages of spore formation and was exclusively found in FP at 25.1 m (0.24%, Figs. 4 and 5). The low relative abundance of spore-forming populations in the older permafrost contrasted with a previous study from the same location showing that the older layers in freshwater permafrost was dominated by spore-forming bacteria [17], but was consistent with other studies of Siberian and Antarctica permafrost [127, 128] as well as permafrost from Svalbard, Norway [129]. Our un-binned metagenome data analysis also showed that metagenomes from 15.0 and 25.1 m had a lower abundance of these genes compared to the top layer samples in FP (Additional file 1: Fig. S5), suggesting that spores were not the most prominent survival strategy in the older freshwater permafrost. The discrepancy between this study and the previous study [17] of freshwater permafrost suggested that the permafrost sampled at this site may be highly heterogeneous, providing large amounts of micro-niches with different environmental characteristics, even from the same borehole.

Since permafrost is frozen, genes encoding cold shock protein (CSPs) were prevalent across the recovered MAGs (38 of 60 MAGs). CSPs are a loosely defined group of DNA binding proteins that are commonly found in cold-adapted microorganisms and were most enriched at 15.0 m in FP (100%), followed by 25.1, 6.0 and 2.95 m (99.76, 67.74 and 62.95%, respectively, Fig. 4). In BP and MP, CSP genes were highest in BP_5.4 (100%, respectively) and lowest in BP_1.75 m (25.46%). The overall high abundance of CSP genes across the samples indicates that microbial communities are well-adapted to stresses associated with freezing temperatures, nutrient starvation and growth deprivation [130].

## Conclusion

This study showed that age, depth and salinity of permafrost sediments shaped biodiversity, community composition and metabolic potential in ancient Siberian permafrost deposits. Microbial communities shifted from Acidobacteriota in the upper younger layers to Gammaproteobacteria and Chloroflexota at the deeper older layers in freshwater borehole AL1_15 and brackish/marine borehole CH1_17, respectively. In addition, genome- and gene-centric analyses revealed that the recovered populations encoded the capacity for hydrolysis, fermentation, dissimilatory nitrate reduction, denitrification, and sulfur reduction (Fig. 6).

Despite many differences between the BP, brackish permafrost and FP, freshwater permafrost, their microbial communities were similar compared with deep old MP, marine permafrost and deep old FP, freshwater permafrost. However, many functions, especially fermentation and nitrogen cycling, could be performed by many members of each of these communities, and were present in all samples. Environmental characteristics, especially temperature, affect nearly every aspect of how microorganisms interact with and are constrained by their environment. An average microbial metabolic rate estimated at permafrost temperatures of −5° to − 10 °C is low and equals 10^–2^–10^–4^ g C h^−1^ [131]. The metabolic processes are susceptible to changes in temperature and metabolic rate, being temperature-dependent, is expected to increase with increasing environmental temperature. With ongoing climate warming a threat of abrupt thawing of glaciers, ground ice, and permafrost increases. In its turn the permafrost thawing will cause a revival of permafrost microbes and trigger decomposition of millennia old carbon stocks. Analyses of permafrost metagenomes unveil taxonomic and metabolic potential of preserved at negative temperatures microbial communities and help to predict their behavior upon permafrost thawing. Although there are large differences in microbial community compositions between young versus old and freshwater versus marine deposits, the functional possibilities for the sampled microbial communities are similar, especially with respect to carbon degradation. All samples exhibited a wide range of taxa capable of fermentation coupled to high energy electron acceptors. The widespread presence of cytochrome *c* oxidase in Siberian permafrost from surface to 25 m depth highlighted aerobically respiring microbial communities well suited for oxygenated soils. Microbial taxa from a wide variety of habitats representing aerobic to anaerobic habitats, as well as freshwater and marine depositional environments and with ages transpiring > 10 kyr years exhibited diverse metabolic potential that could be shaped into diverse aerobic or anaerobic communities under varied physicochemical properties and upon permafrost thawing.

## Supplementary Information

**Additional file 1**. The file contains supplementary figures: **Fig. S1**, Map of sampling sites; **Fig. S2**, Phylogenetic tree of cbbL genes; **Fig. S3**, Abundances of genes involved in methane, nitrogen and sulfur metabolisms; **Fig. S4**, Phylogenetic tree of nifH genes; **Fig. S5**, Abundances of genes involved in motility, bacterial secretion systems and sporulation.

**Additional file 2**. The file contains six Excel tables: **Table S1**, Sample and sequencing information; **Table S2**, Sequence information for MAGs; **Table S3**, Metabolic potential; **Table S4**, Key metabolic pathways for MAGs; **Table S5**, Potential metabolic pathways identifies in MAGs.

## Data Availability

The datasets generated for this study can be found in the NCBI Short Read Archive (SRA) under accession number PRJNA634390 for amplicon sequencing data and under accession number PRJNA601698 for metagenome raw reads.
