# Supplementary material for: Microbial life in 25-m-deep boreholes in ancient permafrost illuminated by metagenomics
Source: Environ Microbiome. 2023 Apr 13;18:33. doi: 10.1186/s40793-023-00487-9 (PMC10103415; doi:10.1186/s40793-023-00487-9)
Supplement: Supplementary file 1 — Additional file 1. The file contains supplementary figures: Fig. S1, Map of sampling sites; Fig. S2, Phylogenetic tree of cbbL genes; Fig. S3, Abundances of genes involved in methane, nitrogen and sulfur metabolisms; Fig. S4, Phylogenetic tree of nifH genes; Fig. S5, Abundances of genes involved in motility, bacterial secretion systems and sporulation. [file 40793_2023_487_MOESM1_ESM.docx]

Microbial life in 25-meter-deep boreholes in ancient permafrost illuminated by metagenomics

Xiaofen Wu^1^, Abraham L. Almatari^1^, Wyatt A. Cyr^1^, Daniel E. Williams^1^, Susan M. Pfiffner^1^, Elizaveta M. Rivkina^2^, Karen G. Lloyd^3^, and Tatiana A. Vishnivetskaya^1,2,3*^

^1^Center for Environmental Biotechnology, University of Tennessee, Knoxville, TN 37996, USA;

^2^Soil Cryology Laboratory, Institute of Physicochemical and Biological Problems in Soil Science, Russian Academy of Sciences, Pushchino, 142290, Russia;

^3^Department of Microbiology, University of Tennessee, Knoxville, TN 37996, USA

*** Corresponding Author:** Tatiana A. Vishnivetskaya, Center for Environmental Biotechnology, University of Tennessee, 1416 Circle Drive, Knoxville, TN 37996-1605, Phone: 865-974-8080, Fax: 865-974-8086, Email: [tvishniv@utk.edu](mailto:tvishniv@utk.edu)

Running Title: Metabolic potential in ancient Siberian permafrost

Keywords: Arctic, permafrost, metagenomics, metagenome-assembled genomes, metabolic potential.

**Originality-Significance Statement**

Knowledge about microbial diversity and metabolism in ancient permafrost is very limited. We studied changes in biodiversity and functional gene distributions with response to age, depth and salinity of Siberian permafrost. We concluded that although there are large differences in microbial community compositions between young vs. old and freshwater vs. marine deposits, the functional possibilities for these communities are similar, especially with respect to carbon degradation.


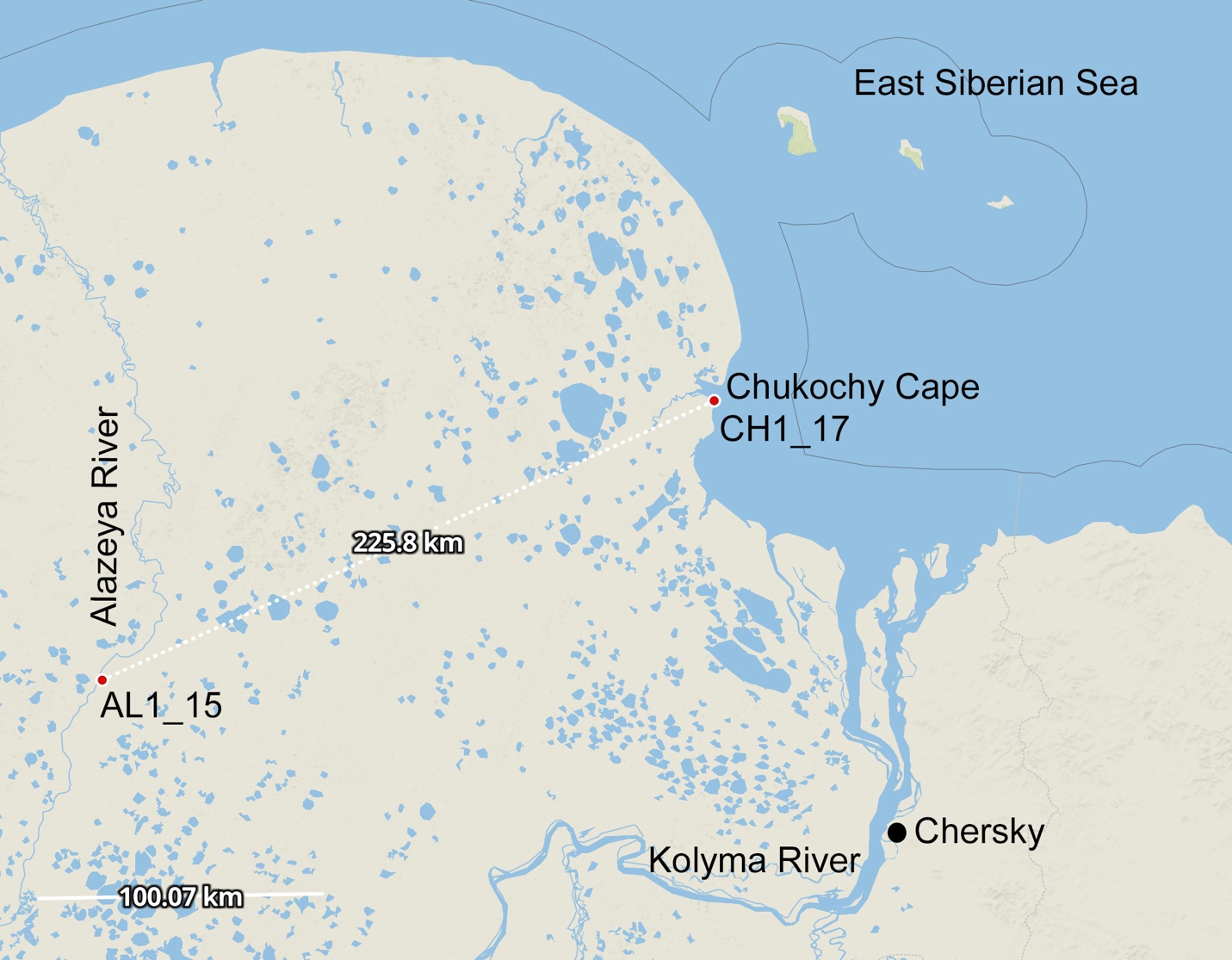


**Fig. S1.** The sampling sites located within the Kolyma Lowland in northeastern Siberia marked with red circles. The first site (AL1_15) was located on the right bank of the Alazeya River (N69º20.438, E154º59.713); the second site (CH1_17) was located at a distance of ~225 km north-east on the Chukochy Cape at the East Siberian Sea coast (N70º04.903, E159º52.282). Borehole AL1_15 was drilled in August 2015 and reached 25.8 m below surface; borehole CH1_17 was drilled in August 2017 and reached 21.7 m below surface. Single borehole was drilled in each site.


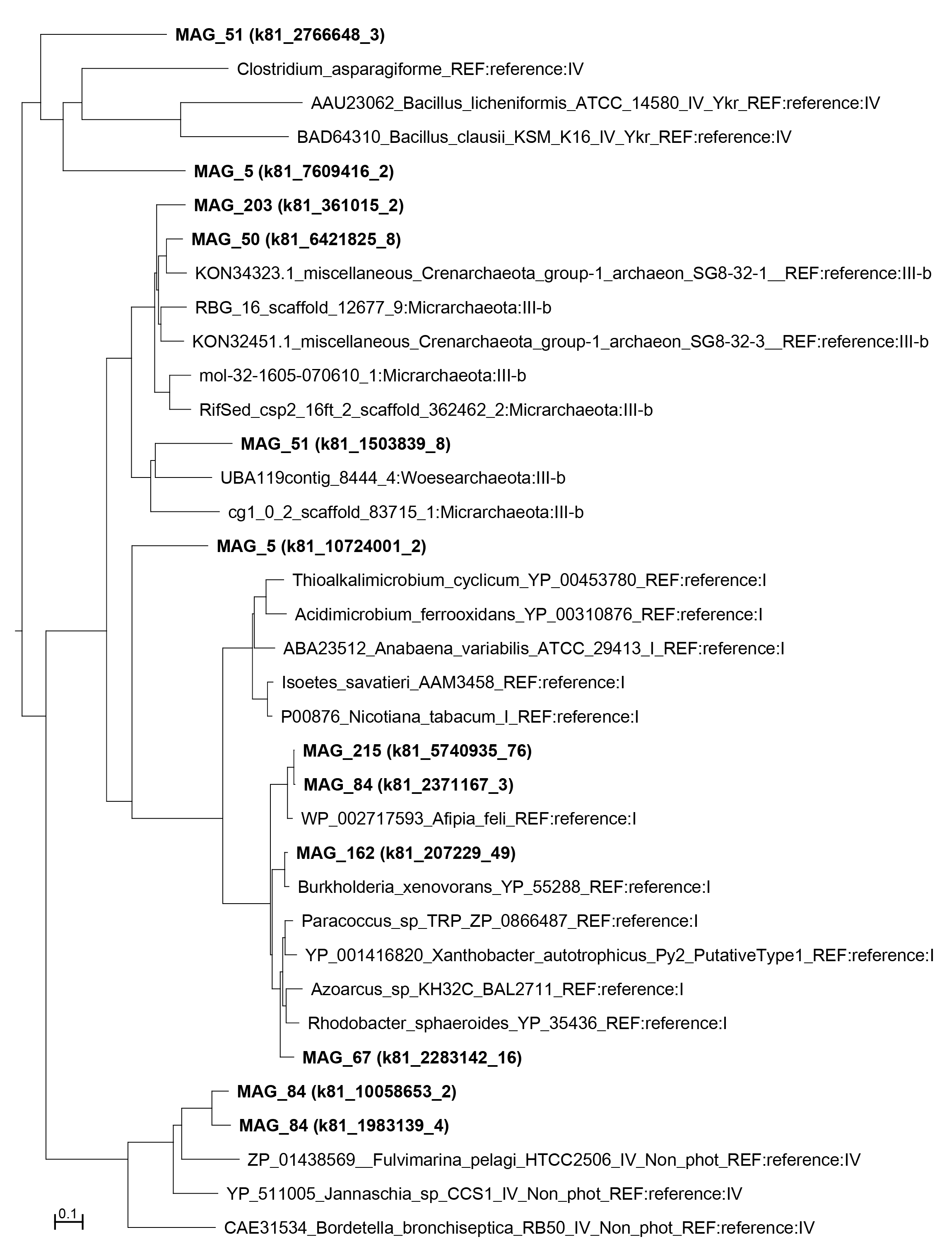


**Fig. S2**. Unrooted neighbor-joining phylogenetic tree of *cbbL* genes. Amino acid sequences of *cbbL* gene extracted from the corresponding MAG from this study are shown in bold. Gene IDs are given in brackets.


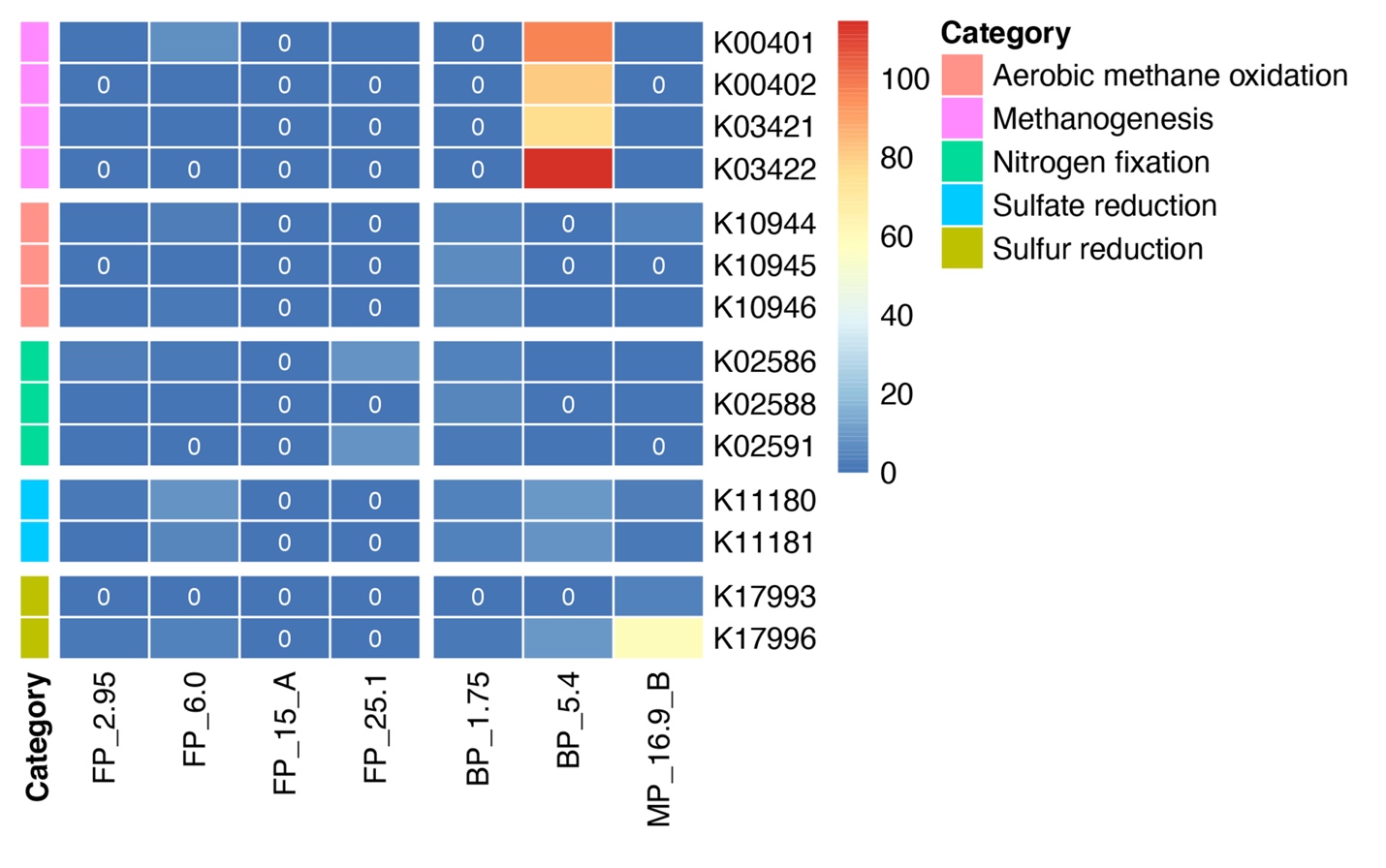


**Fig. S3.** Heatmap showing the normalized abundances of KEGG Orthologies (KOs) involved in methane, nitrogen and sulfur metabolisms. Mean normalized abundances for samples FP_2.95, FP_6.0, FP_25.1, BP_1.75 and BP_5.4 with technical replicates are shown. The 0 written in the dark blue heatmap cells indicates the absence of that gene.

**
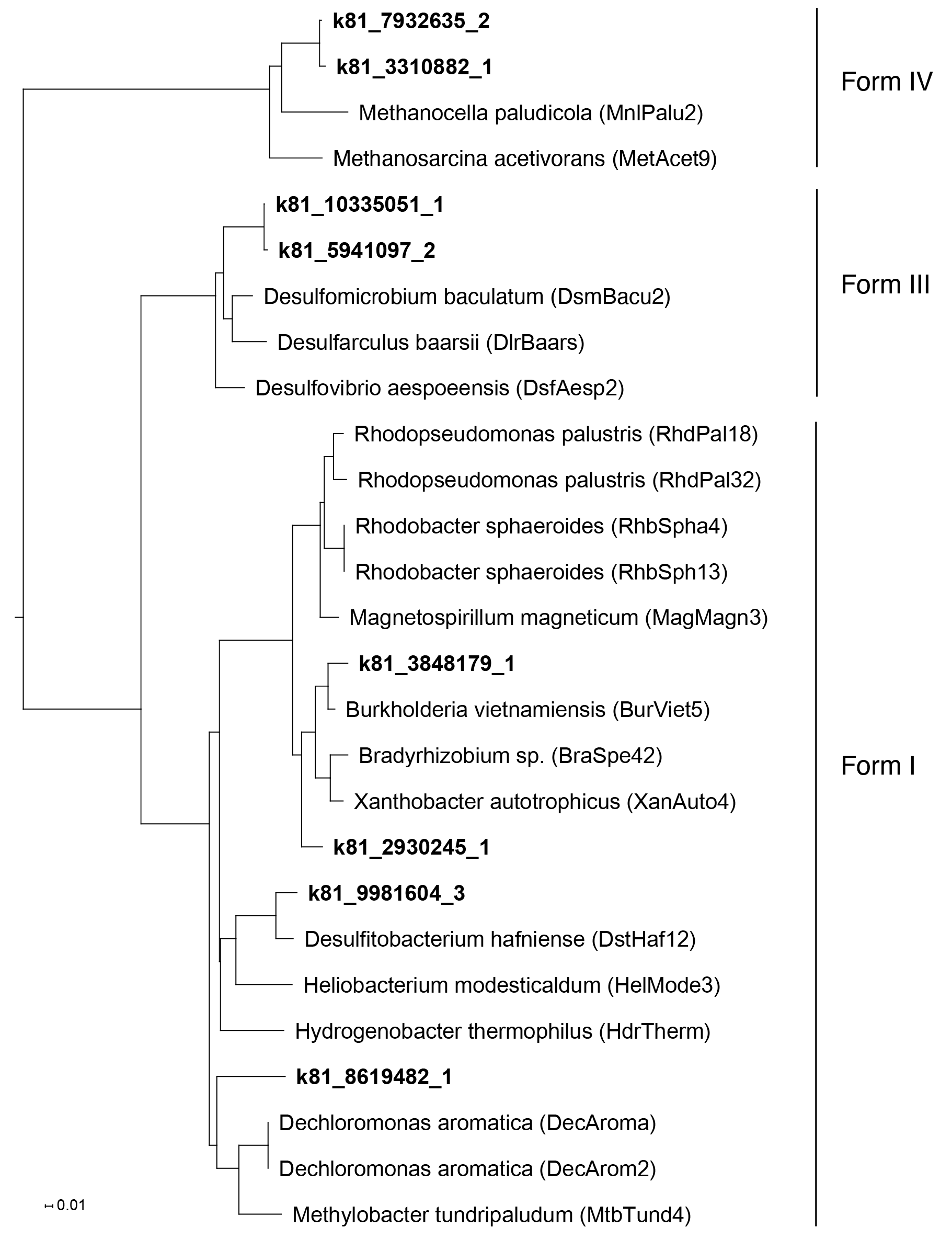
**

**Fig. S4**. Unrooted neighbor-joining phylogenetic tree of *nifH* genes. *nifH* gene obtained from co-assembled contigs are marked in bold.


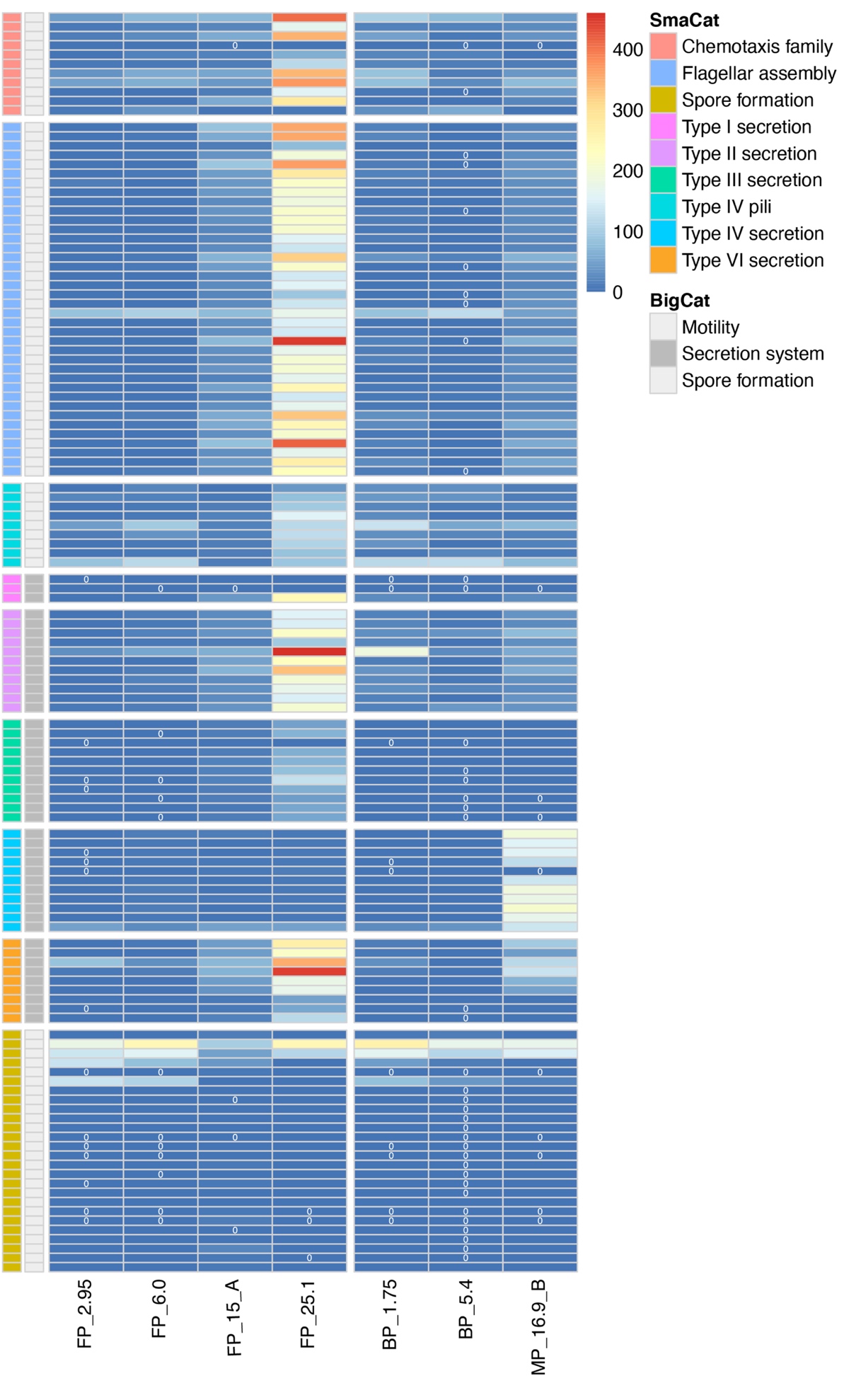


**Fig. S5.** Heatmap showing the normalized abundances of KEGG Orthologies (KOs) involved in motility, bacterial secretion systems and sporulation. Mean normalized abundances for samples FP_2.95, FP_6.0, FP_25.1, BP_1.75 and BP_5.4 with technical replicates are shown. The 0 written in the dark blue heatmap cells indicates the absence of that gene.
